# Structural Analysis of Activities by Japan International Cooperation Agency Volunteer Physiotherapists: Reconstructing Practice-based Knowledge through Text Mining

**DOI:** 10.1298/ptr.25-E10372

**Published:** 2026-02-07

**Authors:** Kosuke HAMADA, Akira TERAMURA, Hyunjae WOO, Akira MITAMURA, Osamu WATANABE

**Affiliations:** 1 Department of Rehabilitation, Aichi Medical College for Physical and Occupational Therapy, Japan; 2 Department of Rehabilitation Science, Osaka Health Science University, Japan; 3 Department of Rehabilitation, Wakayama Professional University of Rehabilitation, Japan; 4 Department of Rehabilitation, Tohoku Medical and Pharmaceutical University Hospital, Japan; 5 Department of Rehabilitation, Teikyo University of Science, Japan

**Keywords:** JICA volunteer physiotherapists, Practice-based knowledge, Text mining, Structural analysis, International cooperation

## Abstract

**Objectives:**

This study structurally analyzed physiotherapists’ activity outcomes dispatched via the Japan International Cooperation Agency (JICA) volunteer program, aiming to develop future strategies for physiotherapy education, practice, and global health engagement.

**Methods:**

We analyzed Reports 1–5 from 123 JICA-dispatched physiotherapists (2014–2024), focusing primarily on the “activity outcomes” section of Report 5 using KH Coder for text mining. Additional variables, such as year of dispatch, gender, host country, geographic region, and number of physiotherapists at the placement site, were extracted. Regions were divided into 5 categories: Asia, Africa, Oceania, Latin America and the Caribbean, and Central Asia and the Middle East. Frequency and hierarchical cluster analyses were also performed. Utilizing clustering patterns, activity outcomes were classified into 8 categories. External variables—region and staffing levels—were used for cross-tabulations.

**Results:**

The study grouped outcomes into 2 main types: 4 categories reflecting direct support through clinical practice and technology transfer, and 3 categories representing institutional and organizational contributions rooted in logical problem solving. The small effect sizes indicated that external factors had limited influence on activity outcomes, such as a greater use of the community-based rehabilitation (CBR) approach in Latin America and areas with limited physiotherapy personnel.

**Conclusions:**

These findings indicate that JICA volunteer physiotherapists consistently fulfilled core professional roles across diverse contexts, emphasizing clinical support, capacity development, and system improvement. Despite regional and contextual differences, the weak effect sizes suggest that individual adaptability and shared professional values contribute to sustaining context-appropriate rehabilitation practices in global health settings.

## Introduction

Recently, the global community has emphasized universal health coverage (UHC) and sustainable development goals (SDGs), making the enhancement and expansion of healthcare services in low- and middle-income countries (LMICs) essential. Within this context, rehabilitation is increasingly recognized in key areas, like disability support, geriatric care, and chronic disease management, where restoring and maintaining functional capacity is essential^1)^.

Physiotherapists have taken center stage in global health discussions. The World Health Organization’s “Rehabilitation 2030” initiative calls for the strategic development of rehabilitation professionals, including physiotherapists, not only in clinical care but also in policy, education, and community outreach^2)^. In LMICs, their role goes beyond individual treatment to include community-based initiatives and promotion of the community-based rehabilitation (CBR) approach in collaboration with health professionals, educators, and public sector stakeholders.

Since 1965, the Japan International Cooperation Agency (JICA) has dispatched over 50000 volunteers to LMICs, including 666 physiotherapists, making them one of the most represented health professionals alongside nurses and midwives^3)^. As of 2024, Japan had the highest number of licensed physiotherapists worldwide, with 213735 practitioners^4)^, underscoring its potential to contribute to international rehabilitation efforts.

JICA volunteer physiotherapists offer flexible, context-sensitive support across areas such as disability rehabilitation, social participation, chronic disease management, human resource development, and team-based care. These efforts have advanced rehabilitation systems in host countries and promoted mutual understanding between Japan and its partner nations^5)^.

While activity reports have documented achievements such as technology transfer, educational initiatives, and support for local health systems, few studies have examined how these outcomes relate to physiotherapists’ professional competencies. Despite the long history and diverse contributions of JICA volunteer physiotherapists, empirical evidence remains scarce regarding how their multifaceted roles—spanning clinical support, education, system improvement, and intercultural facilitation—are structured and interrelated. Previous studies have largely relied on descriptive or qualitative accounts without cross-regional or systematic analysis,^5–11)^. Therefore, this study hypothesizes that JICA volunteer physiotherapists’ activities share common structural features and consistent outcomes across diverse contexts, while recognizing that social and regional characteristics may lead to variations in specific activity outcomes. By analyzing large-scale textual data from volunteer reports, this research aims to provide empirical evidence to address this gap and clarify the underlying structure of their practice-based contributions.

## Methods

### Participants and data source

This study utilized individual reports submitted by physiotherapists dispatched through the JICA volunteer program over a 10-year period, from 2014 to 2024. These reports are completed by each JICA volunteer during their assignment and consist of 5 installments: Report No. 1 describes the placement site’s background and the needs identified at the outset; Report Nos. 2–4 present activity plans and ongoing activity progress; and Report No. 5, completed just before repatriation, contains a comprehensive self-assessment of the volunteer’s activities.

A total of 123 complete sets (Reports Nos. 1–5) were included, and incomplete cases were excluded. Text mining focused on the “activity outcomes” section of Report No. 5. Additional variables, such as year of dispatch, gender, host country, activity region, and number of physiotherapists at the site, were also extracted. The regions were grouped into 5 categories based on geographic characteristics: Asia, Africa, Oceania, Latin America and the Caribbean, and Central Asia and the Middle East.

All data were obtained from printed reports accessed from the JICA library with official permission. This study was approved by the Research Ethics Committee of the Division of Human Sciences, Graduate School of Human Sciences, Osaka University (Approval No. OUKS2363).

### Analytical procedure

The primary analytical target was the “activity outcomes” section in the fifth report. Text mining was conducted using KH Coder (Version 3 Beta.03i) for context analysis^12,13)^.

As KH Coder extracted terms based on morphological analysis, multiword expressions were defined as forced extraction terms. The following terms were specified for forced extraction: “physiotherapy,” “therapeutic exercise,” “JICA volunteer,” “counterpart,” and “disabled people.” Furthermore, synonymous expressions referring to the same concept (e.g., *rehab*. and *rehabilitation*) were standardized and coded as equivalent terms.

Two types of quantitative analyses were conducted: (1) frequency analysis of term occurrences and (2) hierarchical cluster analysis. Hierarchical cluster analysis followed the recommended settings of KH Coder. Specifically, sentences were used as the unit of analysis, the minimum term frequency was set at 30, the minimum number of sentences was 1, Ward’s method was applied for clustering, and the Jaccard index was used to measure distances. Although the “Auto” option in KH Coder was used to generate an initial number of clusters, the final decision to retain 8 clusters was made after examining the dendrogram structure and ensuring conceptual coherence and distinctness among clusters.

Based on the results of hierarchical cluster analysis and the corresponding concordance patterns, the reported activity outcomes were categorized into 8 thematic categories and coded accordingly. Subsequently, cross-tabulation analyses were conducted using KH Coder, with external variables, namely, activity region and number of physiotherapists at the placement site, treated as dependent variables.

To ensure reliability, the researchers—who included authors with JICA volunteer experience and qualitative research experts—repeatedly examined the categorization and coding until they arrived at a consensus.

## Results

### Deployment trends for volunteer physiotherapists

Volunteer physiotherapists were dispatched to 32 countries. The regional distribution was as follows: Asia (38.2%), Latin America and the Caribbean (22.0%), Africa (17.1%), Oceania (12.2%), and Central Asia and the Middle East (10.6%) (Table 1).

**Table 1.** Volunteer deployment and PT staffing by region

Region	Number dispatched (n (%))	Number of physiotherapists at placement sites (n (%))
Asia	47 (38.2)	0: 9 (19.15)
		<5: 24 (51.06)
		≥5: 14 (29.79)
Latin America and the Caribbean	27 (22.0)	0: 8 (29.63)
		<5: 15 (55.56)
		≥5: 4 (14.81)
Africa	21 (17.1)	0: 5 (23.81)
		<5: 11 (52.38)
		≥5: 5 (23.81)
Oceania	15 (12.2)	0: 8 (53.33)
		<5: 6 (40.0)
		≥5: 1 (6.66)
Central Asia and the Middle East	13 (10.6)	0: 11 (84.62)
		<5: 2 (15.38)
		≥5: 0 (0.0)

PT, physiotherapist

Regarding staff composition, in Asia, Latin America and the Caribbean, and Africa, more than half of the placement sites had multiple physiotherapists. In contrast, over 50% of the sites in Oceania, Central Asia, and the Middle East had no assigned physiotherapists (Table 1).

### Extracted terms

Text mining was conducted on the “activity outcomes” section of each volunteer’s fifth report. Term selection and filtering parameters were applied during preprocessing. This process yielded 32189-word tokens and 2662 unique word types.

The 50 most frequently occurring terms are listed in Table 2. The most common was *activity* (257), followed by *patient* (241), *rehabilitation* (226), *physiotherapy* (220), and *perform* (211). Other frequently occurring terms included *implementation* (163), *colleague* (163), *treatment* (135), *instruction* (133), *knowledge* (107), and *technique* (103).

**Table 2.** Top 50 most frequently occurring terms

Term	Frequency	Term	Frequency	Term	Frequency
Activity	257	Patient	241	Rehabilitation	226
Physiotherapy	220	Perform	211	Implementation	163
Colleague	163	Treatment	135	Instruction	133
Knowledge	107	Technique	103	Advancement	90
Placement	90	Volunteer	79	Staff	73
Able to	70	Community	66	Consider	65
Hospital	63	Development	62	Family	58
Continuity	58	Provision	58	Outcome	57
Study	57	Feel	56	Necessary	51
Evaluation	51	Exercise	50	Frequent	45
Content	45	Physician	44	Japan	44
Home visit	44	Clinical care	42	Understanding	42
Environment	41	Facility	41	Information	40
Local	39	Disabled people	38	Observe	38
Method	38	Improvement	38	Daily life	37
Initially	37	Think	37	Month	36
Counterpart	36	Healthcare	35		

### Classification of outcomes based on cluster analysis

**Fig. 1.**
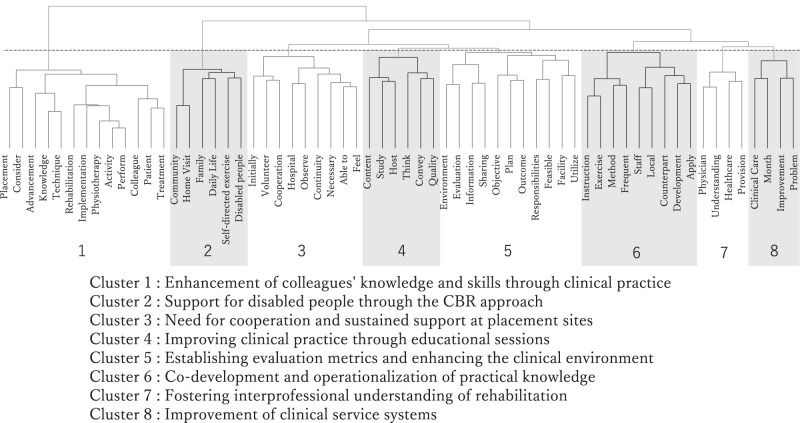
Structure of hierarchical clustering and cluster labels. This figure presents a dendrogram illustrating the distances between each word. In this cluster analysis, Ward’s method was employed to minimize variance during clustering. The distance between words was defined using the Jaccard coefficient. Cutting the dendrogram at the height of the dashed line shows that the words below it were separated into groups.

Hierarchical cluster analysis was performed on lexical items from the “activity outcomes” section of the fifth reports submitted by physiotherapists in the JICA volunteer program. Eight distinct clusters were extracted and labeled based on the contextual relevance of the key terms (Fig. 1).

Cluster 1 included terms—such as *placement, consider, advancement, knowledge, technique, rehabilitation, implementation, physiotherapy, activity, perform, colleague*, *patient*, and *treatment*—which reflected daily clinical practice and on-the-job education. This cluster was labeled “Enhancement of colleagues’ knowledge and skills through clinical practice,” underscoring the physiotherapists’ roles in transferring expertise.

Cluster 2 featured terms—such as *community*, *home visit*, *family*, *daily life*, *self-directed exercise*, and *disabled people*—indicating a CBR context in which support was extended beyond institutions to homes and communities. This cluster was labeled “Support for disabled people through the CBR approach.”

Cluster 3 contained terms—such as *initially*, *volunteer*, *cooperation*, *hospital*, *observe*, *continuity*, *necessary*, *able to*, and *feel*—reflecting persistent challenges in building sustainable relationships and support systems. This cluster was labeled “Need for cooperation and sustained support at placement sites.”

Cluster 4 was centered on terms—such as *content, study, host, think, convey*, and *quality*—indicating activities such as workshops and training aimed at enhancing staff competence and information sharing. This cluster was labeled “Improving clinical practice through educational sessions.”

Cluster 5 included *environment, evaluation, information, sharing, objective, plan, outcome, responsibilities, feasible, facility*, and *utilize*—suggesting the implementation of evaluation tools and improvements in clinical or workplace settings. This cluster was labeled “Establishing evaluation metrics and enhancing the clinical environment.”

Cluster 6 featured terms—such as *instruction, exercise, method, frequent, staff, local, counterpart, development,* and *apply*—pointing to the joint creation and application of clinical resources such as manuals and assistive tools. This cluster was labeled “Co-development and operationalization of practical knowledge.”

Cluster 7 included terms—such as *physician*, *understanding*, *healthcare*, and *provision*—indicating interdisciplinary collaboration and efforts to promote a shared understanding of rehabilitation. This cluster was labeled “Fostering interprofessional understanding of rehabilitation.”

Cluster 8 comprised terms—such as *clinical care, month*, *improvement*, and *problem*—highlighting efforts to identify and address recurring challenges in monthly clinical operations. This cluster was labeled “Improvement of clinical service systems.”

### Frequency analysis of activity outcomes and associations with external variables

The activity outcomes were categorized by establishing coding rules based on cluster-specific terms. Each report was coded according to the relevant terms associated with each cluster. The frequencies of each code were then calculated (Table 3).

**Table 3.** Simple aggregation of activity outcomes

Category name	Frequency	Percentage
*Enhancement of colleagues’ knowledge and skills through clinical practice*	821	78.3
*Need for cooperation and sustained support at placement sites*	350	33.4
*Co-development and operationalization of practical knowledge*	336	32.0
*Establishing evaluation metrics and enhancing the clinical environment*	285	27.2
*Improving clinical practice through educational sessions*	186	17.7
*Support for disabled people through the CBR approach*	181	17.3
*Fostering interprofessional understanding of rehabilitation*	151	14.4
*Improvement of clinical service systems*	125	11.9
# No applicable code	63	6.0
Total number of segments	1049	

The most frequently observed category was “Enhancement of colleagues’ knowledge and skills through clinical practice” (78.3%), followed by “Need for cooperation and sustained support at placement sites” (33.4%) and “Co-development and operationalization of practical knowledge” (32.0%). Other categories included “Establishing evaluation metrics and enhancing the clinical environment,” “Improving clinical practice through educational sessions,” “Support for disabled people through the CBR approach,” “Fostering interprofessional understanding of rehabilitation,” and “Improvement of clinical service systems.”

To investigate associations with external variables, cross-tabulations and chi-squared (χ^2^) tests were conducted. The external variables were as follows: (1) geographic region of assignment and (2) number of physiotherapists at the placement site (Tables 4 and 5).

**Table 4.** Regional distribution of activity outcomes

Category	Asia	Africa	Oceania	Central Asia Middle East	Latin America	χ^2^ (df)	*p*-Value	Cramér’s V
Support via the CBR approach	15.55%	7.59%	19.01%	19.64%	**24.58%**	20.91 (4)	<0.01	0.14
Understanding among professionals	14.11%	18.35%	20.66%	**21.43%**	5.83%	24.66 (4)	<0.01	0.15
Practice-based knowledge formalization	29.67%	33.54%	**43.80%**	28.57%	32.02%	9.71 (4)	<0.05	0.09

Percentages indicate the proportion of coded responses per region.

Only categories with statistically significant differences are presented.

Statistical significance was assessed using the chi-squared test.

Adjusted standardized residuals (not shown) indicate regional overrepresentation (≥+1.96) or underrepresentation (≤−1.96)

Bold underlining indicates values with adjusted standardized residuals ≥ +1.96 and higher-than-expected frequencies.

Underlining indicates values with adjusted standardized residuals ≤ −1.96 and lower-than-expected frequencies.

CBR, community-based rehabilitation

**Table 5.** Activity outcomes by number of physiotherapists at host institutions

Category	PTs = 0	PTs <5	PTs >5	χ^2^ (df)	*p*-Value	Cramér’s V
Support via the CBR approach	**21.20%**	18.76%	6.57%	20.43 (2)	<0.01	0.14

Percentages indicate the proportion of coded responses per region.

Only categories with statistically significant differences are presented.

Statistical significance was assessed using the chi-squared test.

Adjusted standardized residuals (not shown) indicate regional overrepresentation (≥+1.96) or underrepresentation (≤−1.96)

Bold underlining indicates values with adjusted standardized residuals ≥ +1.96 and higher-than-expected frequencies.

Underlining indicates values with adjusted standardized residuals ≤ −1.96 and lower-than-expected frequencies.

PT, physiotherapist; CBR, community-based rehabilitation

First, the outcomes were cross-tabulated based on region (Asia, Africa, Oceania, Central Asia and the Middle East, and Latin America and the Caribbean), and χ^2^ tests were applied. The 3 categories exhibited significant regional differences:

• *Support for disabled people through the CBR approach* (χ^2^(4) = 20.91, *p* <0.01, V = 0.14)
• *Fostering interprofessional understanding of rehabilitation* (χ^2^(4) = 24.66, *p* <0.01, V = 0.15)
• *Co-development and operationalization of practical knowledge* (χ^2^(4) = 9.71, *p* <0.05, V = 0.09)

Although statistically significant, the effect sizes (Cramér’s V) ranged from weak to moderate. No other categories showed significant regional variations.

A similar analysis based on physiotherapist numbers at the placement site (0, <5, ≥5) revealed only one significant result:

• *Support for disabled people through the CBR approach* (χ^2^(2) = 20.43, *p* <0.01, V = 0.14)

Likewise, the association’s strength ranged from weak to moderate. No other major differences were identified based on the staffing levels.

## Discussion

### Key findings and contribution

This study makes 4 main contributions. First, using large-scale text mining (32189 tokens; 2662 types), we empirically derived an 8-category framework that reconstructs volunteer physiotherapists’ practice-based knowledge beyond a list of activities. Second, we proposed a 2-axis model that differentiates direct professional contributions (clinical practice and technology transfer) from system-level/organizational support grounded in logical improvement. Third, cross-tabulations showed small effect sizes (Cramér’s V: 0.09–0.15), indicating that structural consistency of roles and outcomes predominated across regions and staffing contexts—consistent with our hypothesis. Fourth, we identified context-specific tendencies aligned with local needs—greater use of CBR in Latin America and at sites with few/no physiotherapists, more co-development of practical knowledge in Oceania, and stronger interprofessional understanding initiatives in Central Asia and the Middle East—thereby demonstrating that limited but meaningful contextual variation coexists with overall consistency.

### Conceptual reconstruction of volunteer physiotherapists’ practical knowledge

Frequent term analysis revealed that basic terms like “activity,” “patient,” “rehabilitation,” and “physiotherapy” ranked among the most common, reaffirming that clinical practice forms the core of volunteer engagement. This supports prior findings indicating that the primary role of volunteer physiotherapists is to provide therapeutic exercises to disabled people within institutional settings^14)^. Meanwhile, the frequent appearance of terms such as “instruction,” “knowledge,” “skills,” and “colleague” indicates indirect support and educational contributions to local staff, beyond direct service delivery. This suggests that volunteers play a broader role in fostering problem-solving capacity, enabling local personnel and organizations to independently identify issues and work toward achieving their goals. In the global healthcare domain, the importance of human resource development and “capacity building” has already been recognized^15,16)^, and this study’s findings reinforce these perspectives.

A hierarchical cluster analysis identified 8 outcome categories based on the patterns of co-occurring terms found in the reports. These categories go beyond a list of implemented activities, offering a conceptual structure of practical knowledge that reflects physiotherapists’ multifaceted contributions to local healthcare and rehabilitation systems, spanning education, technology transfer, institutional support, and interprofessional collaboration. Excluding the category “Need for cooperation and sustained support at placement sites,” the remaining outcomes can be organized into 2 primary axes: (1) direct professional contributions involving the application of physiotherapy expertise and technology transfer, and (2) system-level or organizational support grounded in logical analysis and improvement practices, drawing on the perspective of Japanese healthcare professionals.

Although host countries often request volunteer physiotherapists mainly for technical transfer, they are frequently relied upon to address chronic human resource shortages. Nonetheless, individual volunteers effectively analyzed local issues and generated multifaceted outcomes, including institutional and organizational improvements. Such problem-solving-oriented practices demonstrate that physiotherapists possess not only technical expertise but also the ability to comprehensively understand and address the host communities’ broader needs.

### Consistent activities and outcomes across diverse social contexts

The results of this study revealed that external factors, such as the number of physiotherapists at host institutions and the regions of assignment, did not significantly influence activity outcomes. Rather, despite the wide variation in the social contexts and periods of assignment, the essential roles and activity outcomes of volunteer physiotherapists remained consistent.

Five categories—“Need for cooperation and sustained support at placement sites,” “Enhancement of colleagues’ knowledge and skills through clinical practice,” “Improving clinical practice through educational sessions,” “Establishing evaluation metrics and enhancing the clinical environment,” and “Improvement of clinical service systems”—demonstrated particularly strong consistency, independent of external conditions. This finding indicates that the core responsibilities of volunteer physiotherapists revolve around the provision of rehabilitation services, capacity building for local staff, and the clinical operations management.

In particular, activities classified under “Improvement of clinical service systems” included the development of standardized patient records, the introduction of clinical assessment tools, and the creation or revision of clinical protocols. These initiatives strengthened care continuity, boosted information exchange among staff, and supported sustainable improvements tailored to local medical cultures and institutional frameworks. Such initiatives were rarely completed within a single volunteer term but were often handed over to successor volunteers with the host institution’s support, thereby contributing to cumulative progress.

At the same time, certain tendencies were observed according to regional contexts and placement environments. The category “Co-development and operationalization of practical knowledge” appeared most frequently in Oceania, while “Support for disabled people through the CBR approach” was commonly found in Latin America and in sites with few or no physiotherapists. “Fostering interprofessional understanding of rehabilitation” was most frequent in Central Asia and the Middle East. However, these differences exhibited small effect sizes statistically, suggesting that they reflected cultural and institutional variations rather than true regional disparities. These results reaffirm the study hypothesis that volunteer physiotherapists’ activities maintain structural consistency across contexts while adapting to social and regional conditions.

The category “Co-development and operationalization of practical knowledge” represented collaborative practices in which volunteers and local staff jointly created context-specific manuals, exercise guides, and assistive tools, rather than engaging in 1-way skill transfer. These practices align with the global health principles of appropriate technology and sustainable, locally responsive support^17)^.

The category “Support for disabled people through the CBR approach” indicated that in environments with scarce rehabilitation resources, including human resources, providing specialized technical education was often challenging. Instead, a comprehensive, community-wide support model was required. This finding is consistent with previous studies identifying the CBR approach as an effective strategy for disability support in resource-limited settings^18)^. CBR is frequently described as the “democratization of rehabilitation,” aiming to ensure that everyone can access and participate in rehabilitation services^19)^. From this perspective, volunteer physiotherapists contribute to improving access to rehabilitation in underserved communities. However, it is important to note that the term “CBR” encompasses a wide range of interventions and is interpreted differently across countries. In some cases, it overlaps with Japan-specific practices such as home-based or community rehabilitation. Therefore, the value of such activities cannot be uniformly evaluated.

The category “Fostering interprofessional understanding of rehabilitation” was primarily observed in regions where physiotherapists were few or absent, suggesting that volunteers often engaged in advocacy to raise professional recognition. The lack of recognition for physiotherapists is reflected in World Physiotherapy membership status, where Central Asian countries are not members and Middle Eastern representation remains limited^20)^.

Nevertheless, the low Cramér’s V values obtained in all analyses indicate that the influence of external factors—namely, the number of physiotherapists and the region of deployment—on activity outcomes was minimal. Therefore, the central implication of this study is not regional differences but rather the structural uniformity and shared challenges characterizing volunteer physiotherapists’ activities across diverse social contexts.

The underlying issues faced by the countries and regions that request volunteer physiotherapists are fundamentally similar despite differences in culture, religion, and socioeconomic background. Accordingly, activities have developed in similar directions within their respective contexts. Moreover, as volunteers conduct their activities with reference to Japan’s healthcare, long-term care, and welfare systems, they may identify shared challenges that reflect the collective perspectives of Japanese physiotherapists.

Although volunteer physiotherapists are expected to tailor their activities and outcomes to local cultures, environments, staffing compositions, and community needs, their roles and contributions in resource-limited settings demonstrate a generally consistent direction. This suggests that the practical knowledge accumulated through their experiences functions as transferable expertise that transcends regional and temporal boundaries.

These insights furnish both theoretical and practical foundations for physiotherapists and other rehabilitation professionals involved in international cooperation, serving as valuable resources for future practices and policy formulation.

### The broader significance of physiotherapist engagement in global health

This study highlights that physiotherapists dispatched through the JICA volunteer program contribute beyond clinical support. In many LMICs with few rehabilitation professionals and limited services, volunteers implement context-specific CBR, promote interdisciplinary collaboration, and introduce appropriate technologies^21)^. These actions reflect their roles as flexible and comprehensive support agents addressing institutional and cultural gaps. Such international experiences offer professional growth opportunities not available in domestic settings, enabling physiotherapists to expand their skills and engage in reciprocal learning.

As global health issues become more complex, they necessitate multidisciplinary solutions. Professionals are needed to “translate” local needs into contextually appropriate interventions.

Physiotherapists, with their broad expertise and adaptability, are well-suited to this role. Through international cooperation, they refine their expertise in diverse cultural and systemic contexts, working alongside local partners to transform both care delivery and outcomes. In this way, physiotherapy can transcend traditional roles by enabling physiotherapists to comprehensively understand the challenges faced by host communities and develop context-appropriate, sustainable solutions.

The outcomes identified in this study point to the vision of physiotherapists not merely as technical providers but also as autonomous, innovative agents of support. Global health initiatives serve as a platform for cultivating professional skills and humanistic values, inspiring a new generation that is committed to meaningful international engagement.

### Limitations and directions for future research

This study had several limitations. First, the primary data sources were self-reported narratives, which are inherently subjective and may emphasize positive outcomes. Second, text mining involves interpretive decisions regarding word selection and coding, which potentially affect replicability. Moreover, this method may not fully capture the contextual depth of narrative content.

Future research should incorporate triangulated sources, such as interviews and site evaluations, to validate these results. Developing qualitative evaluation frameworks may further support the objective assessment of the institutional and social impacts of volunteer physiotherapists.

Nevertheless, this study has several strengths. It is the first to quantitatively and structurally analyze a decade of JICA volunteer physiotherapists’ reports using text mining, providing empirical insights into their multifaceted roles across diverse contexts. The integration of qualitative interpretation and quantitative analysis enhances the reliability and applicability of the findings for future research and policy development.

## Conclusions

This study presents a structured framework for understanding the activities and outcomes of volunteer physiotherapists, providing valuable insights that can inform and advance future research and practice in the field of international cooperation.

## References

[1] World Health Organization. The World Bank: Tracking universal health coverage: 2017 global monitoring report. https://apps.who.int/iris/bitstream/handle/10665/259817/9789241513555-eng.pdf (Accessed May 1, 2025).

[2] World Health Organization: Rehabilitation 2030 initiative 2017. https://www.who.int/initiatives/rehabilitation-2030 (Accessed May 1, 2025).

[3] Japan International Cooperation Agency: Project and dispatch achievements. https://www.jica.go.jp/volunteer/outline/publication/results/index.html (in Japanese) (Accessed May 1, 2025).

[4] Japanese Physical Therapy Association: Statistical information. https://www.japanpt.or.jp/activity/data/. (in Japanese) (Accessed May 1, 2025).

[5] Chiwaki N: Kokusai kyōryoku bunya de no rigaku ryōhōshi, sagyō ryōhōshi katsudō no kōka kenkyū. Phys Ther Jpn. 2016; 43: 160–161. (in Japanese)

[6] Namura H: Report of Japan Overseas Cooperation Volunteers activities: looking back on the volunteer activities in Vietnam. Sci Res Phys Ther. 2013; 4: 31–39. (in Japanese)

[7] Aizawa E: JICA’s approach to disability and development: expectations for physical therapists. Phys Ther Jpn. 2015; 42: 655–656. (in Japanese)

[8] Nakao G, Corneliaus M, et al.: The work of a Japan International Cooperation Agency volunteer as a physiotherapist in Fiji. Sapporo J Health Sci. 2021; 10: 57–61.

[9] Hamada K, Teramura A: The situation of rehabilitation in Honduras. Jpn J Phys Ther. 2021; 55: 484–485. (in Japanese)

[10] Mitamura A: Fiji Kyōwakoku ni okeru JICA kaigai kyōryokutai no keiken to kokusai jigyō katsudō no shōkai. Annu Rep Miyagi Phys Ther Assoc. 2022; 33: 28–37. (in Japanese)

[11] Koizumi Y: Sekai ni me o mukeru rigaku ryōhōshi to kokusai kyōryoku. Phys Ther Skill Res. 2024; 52: 9–12. (in Japanese)

[12] Higuchi K: Shakai chōsa no tame no keiryō tekisuto bunseki: naiyō bunseki no keishō to hatten o mezashite: KH Coder official book. 2nd ed, Nakanishiya Shuppan, Kyoto, 2020, 120-227. (in Japanese)

[13] Higuchi K: A two-step approach to quantitative content analysis: KH Coder tutorial using Anne of Green Gables (Part I). Ritsumeikan Soc Sci Rev. 2016; 52: 77–91.

[14] Hamada K, Shimizu K, et al.: Role of volunteer physiotherapists in the field of international cooperation: a qualitative analysis of JICA volunteer requests. J Aichi Soc Phys Ther. 2024; 36: 112–120. (in Japanese)

[15] World Health Organization: Capacity building and initiatives. In: Health Promotion. https://www.who.int/teams/health-promotion/tobacco-control/implementing/capacity-building (Accessed June 6, 2025).

[16] Frenk J, Chen L, et al.: Health professionals for a new century: transforming education to strengthen health systems in an interdependent world. Lancet. 2010; 376: 1923–1958. 21112623 10.1016/S0140-6736(10)61854-5

[17] Watanabe M: Activities of Physical Therapists in JICA Volunteer. Phys Ther Jpn. 2015; 42: 657–658. (in Japanese)

[18] World Health Organization, The World Bank: World report on disability 2011. https://www.who.int/teams/noncommunicable-diseases/sensory-functions-disability-and-rehabilitation/world-report-on-disability (Accessed June 6, 2025). 26131540

[19] Helander E, Mendis P, et al.: Training in the community for people with disabilities. World Health Organization, Geneva, 1989. https://iris.who.int/bitstream/handle/10665/39065/31922_intro.pdf (Accessed June 6, 2025).

[20] World Physiotherapy: Member organizations in Asia Western Pacific region. https://world.physio/ja/regions/asia-western-pacific (Accessed June 19, 2025).

[21] Kuno K: Korekara no rehabilitation: WHO Rehabilitation 2030 kaigi kara. Jpn J Phys Ther. 2019; 53: 977–984. (in Japanese)

